# Special electromagnetic field-treated water and far-infrared radiation alleviates lipopolysaccharide-induced acute respiratory distress syndrome in rats by regulating haptoglobin

**DOI:** 10.1080/21655979.2021.1969201

**Published:** 2021-09-14

**Authors:** Changyong Luo, Yan Li, Xu Liang, Yifan Chen, Qiao Zou, Yurong Kong, Zhengguang Guo, Wei Sun, Xin Wang

**Affiliations:** a Infectious fever center, Dongfang Hospital of Beijing University of Chinese Medicine, Beijing, China; b Education section, Dongzhimen Hospital of Beijing University of Chinese Medicine, Beijing, China; c The graduate school, Beijing University of Chinese Medicine, Beijing, China; d Central laboratory, Institute of Basic Medical Sciences, Academy of Medical Science, Peking Union Medical College, Beijing, China; e Research institute, Biological Spectrum Institute, Guangdong Junfeng BFS Technology CO, Guangdong, China

**Keywords:** Special electromagnetic field-treated water, far-infrared radiation, lipopolysaccharide, haptoglobin, acute inflammatory response

## Abstract

Special electromagnetic field-treated water (SEW) and far-infrared radiation (FIR) can reduce acute respiratory distress syndrome (ARDS) in rats inflicted by lipopolysaccharides (LPSs). However, little is known about its underlying molecular mechanism. Differentially expressed proteins (DEPs) of SEW and FIR interventions were obtained from a proteomics database. A total of 89 DEPs were identified. Enrichment analysis of DEPs was performed using the Database for Annotation, Visualization, and Integrated Discovery. These DEPs were associated with the responses to LPSs, acute inflammation, extracellular exosomes, glucocorticoids, and electrical stimuli. The protein-protein interaction network was set up using the STRING database. Modular analysis was performed using MCODE in the Cytoscape software. Proteins Haptoglobin, Apolipoprotein B, Transthyretin, and Fatty acid binding protein 1 were among the core networks. A tail vein injection of LPS was used to establish the rat model with ARDS. Parallel reaction monitoring confirmed Hp protein expression. Inflammatory pathway factors were detected using an enzyme-linked immunosorbent assay. This indicates that SEW and FIR can be considered as potential clinical treatment methods for ARDS treatment and that their functional mechanisms are related to the ability of alleviating lung inflammation through Hp protein adjustment.

## 1 Introduction

Acute respiratory distress syndrome (ARDS) occurs due to a combination of multiple intrapulmonary and extrapulmonary causes with respiratory distress and progressive hypoxemia as the main clinical manifestations **[**1**]**, which have attracted substantial attention due to its high mortality rate. The mortality rate of patients with severe ARDS is as high as 40% or more **[**2**]**. The coronavirus disease 2019 pandemic has caused an increase in ARDS and highlighted challenges associated with this syndrome **[**3**]**. Scientists are constantly trying new ways to explore the pathogenesis of ARDS, such as consensus analysis via weighted gene co-expression network analysis, revealing genes participating in the early phase of sepsis-induced ARDS **[**4**]**. Several researchers have confirmed that it is an excessive inflammatory reaction mediated by cells and body fluids on the alveolar capillaries, and its development process can be summarized as the migration and aggregation of inflammatory cells and excessive release of inflammatory mediators, which in turn leads to an increase in alveolar permeability **[**5**]**. In the United States, approximately 200,000 patients suffer ARDS each year, causing nearly 75,000 deaths, which have exceeded breast cancer or human immunodeficiency virus infection **[**6**]**.

**Figure 1.**
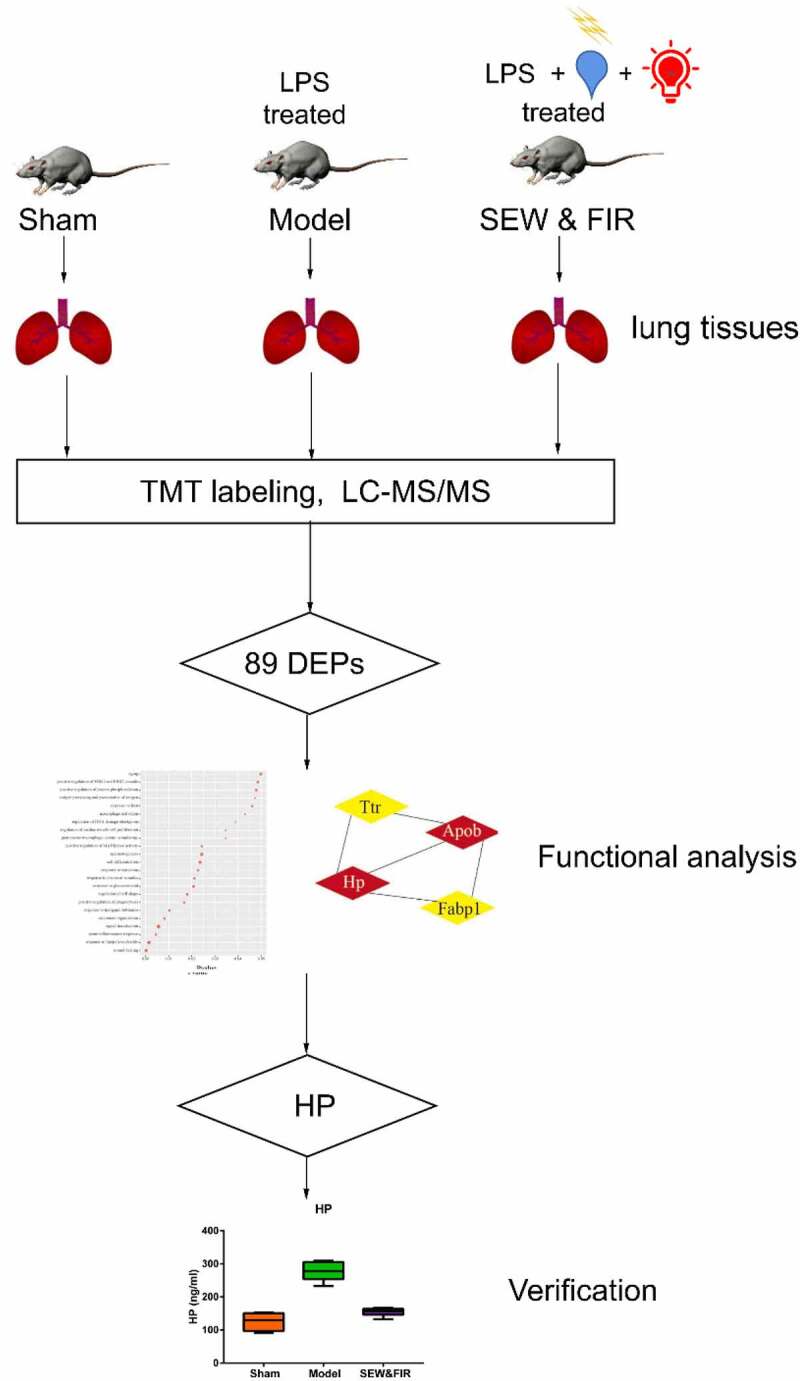
The workflow of the study

Special electromagnetic field-treated water (SEW) is a type of functional water treated using an ultralow-frequency magnetic field, that is, the physical and chemical characteristics of ordinary drinking water have been changed, such as smaller water molecular clusters and higher dielectric constants **[**7**]**. The safety evaluation experiment on SEW found that SEW is safe and nontoxic and can improve the body’s immunity **[**8**]**. As a type of physical therapy, far-infrared radiation (FIR) can improve local blood circulation, strengthen tissue metabolism, promote swelling subsidence, and treat inflammatory diseases **[**9**]**. FIR may promote wound site healing by stimulating transforming growth factor-beta secretion or fibroblast activation **[**10**]**. Previously, we demonstrated that SEW and FIR can significantly alleviate lung and tissue damage by decreasing inflammatory cell infiltration **[**11**]**. Experiments revealed that the protective effect of SEW and FIR on endotoxin-induced ARDS may result from their role in reducing the levels of interleukin (IL)-1β and IL-6 in serum and the expression level of nuclear factor kappa-B (NF-κB) signaling pathways in lung tissue **[**12**]**. Other studies have reported that SEW and FIR have protective effects on lipopolysaccharide (LPS)-induced ARDS in rats, which may be related to the increased expression of IL-4 in serum **[**13**]**. As a physical intervention method, SEW and FIR can interfere with biochemical mechanisms in vivo in many ways. To comprehensively analyze the therapeutic mechanism of SEW and FIR, we used proteomic methods to screen the different proteins among groups, enrich and analyze the different biological functions, and explore the potential effects of SEW and FIR, followed by validation of key proteins with significantly different biological functions to further clarify some of the mechanisms underlying SEW and FIR. Generally, we used proteomics data combined with in vivo experiments to verify the mechanism of SEW and FIR in preventing and treating LPS-induced ARDS in rats (Figure 1).

## 2 Materials and methods

### 2.1 Data preparation and processing

The proteomics data file was downloaded from the proteomics database website (www.iprox.cn) using the data ID: IPX0002375000. The lung tissue was cleaved into peptides, which were further purified and quantified. 8 standard reagent was used for dark incubation at marked room temperature. Finally, one-dimensional LC-MS analysis was performed on the vacuum dried samples as previous published [14]. Data used in this study were the proteomic expression data of the lung tissues of the sham, model, and SEW and FIR groups (6 rats in each group). R Studio version 3.6.2 was used to calculate the differential proteins and to draw the figures. The fold change that meets the model/sham group <0.833, SEW and FIR/model group >1.1, or model/sham group >1.2, and SEW and FIR/model group <0.909 are differentially expressed proteins (DEPs).

### 2.2 Gene Ontology (GO) analysis and protein-protein interaction (PPI) network module analysis

The Database for Annotation, Visualization and Integrated Discovery (DAVID) (https://david.ncifcrf.gov/) was used for gene function enrichment and pathway analysis of differential proteins, and P value scoring was performed according to the degree of correlation between differential proteins and biological functions or pathways, with R language for graphic display. The PPI network of DEPs was constructed using the STRING 11.0 (http://string-db.org/) tool, and the core of the protein network was analyzed using the MCODE function of Cytoscape 3.5.1.

### 2.3 Experimental animals

In total, 18 6-week-old male Sprague-Dawley rats with an average weight of 180 ± 10 g were provided by Beijing Huafukang Biotechnology Co., Ltd., with license no. SCXK (Beijing) 2019–0008. The rats were maintained at the Barrier Laboratory of the Experimental Animal Center of Dongzhimen Hospital of Beijing University of Chinese Medicine. During the experiment, the laboratory temperature was set between 22°C and 24°C, and the humidity was fixed between 50% and 70%, whereas the same standard of ordinary pellet feed was given to the rats while feeding in separate cages. All animal experiments in this study were conducted in accordance with the relevant guidelines and regulations and were approved by the Experimental Animal Welfare and Ethics Committee of Dongzhimen Hospital of Beijing University of Chinese Medicine (ID: 19–54). Moreover, all experimental animals were used and treated to minimize pain.

### 2.4 Main reagents and instruments

The following reagents were used: *Escherichia coli* LPS (Sigma L6511), rat haptoglobin (HP) ELISA Kit (Jiangsu Proteome Biotech Co., Ltd., MB-1920A), rat IL-1β ELISA kit (Jiangsu Proteome Biotech Co., Ltd., MB-1588A), and rat IL-6 ELISA Kit (Jiangsu Proteome Biotech Co., Ltd., MB-1731A). A BH2 biological microscope (Olympus, Tokyo, Japan) and LTQ Orbitrap Fusion Lumos and Easy-n LC 1000 upgraded liquid chromatography (Thermo Scientific, USA) were utilized. The preparation instrument for SEW was Junfeng BFS water treatment and healthcare device JF-118B, whereas that for FIR is Junfeng BFS treatment & healthcare device JF-802.

### 2.5 Experimental animal grouping

The experimental animals were randomly divided into three groups with six animals in each group: the sham, model, and SEW and FIR groups, which were weighed and recorded daily. The sham and model groups were administered with distilled water at a daily volume of 1 mL/100 g, whereas the SEW and FIR group was administered with SEW at a daily volume of 1 mL/100 g and irradiated with FIR for 30 min. On day 7 after 6 h of intragastric administration, the sham group was injected with 2 mg/kg normal saline, whereas the model and SEW and FIR groups were injected with 2 mg/kg LPS solution through the tail vein. After 16 h, the rats in all three groups were allowed to fast for 8 h and dissected under anesthesia. The animals were euthanized by an intraperitoneal injection of 50 mg/kg pentobarbital sodium. The ARDS animal model was administered with LPS through a single intravenous injection to induce a systemic inflammatory response, which is the most common cause of ARDS. The phenotype of this animal model is consistent with the pathological features of ARDS lung tissue, and the modeling method is stable [14].

### 2.6 Pathological observation of lung tissue

Hematoxylin-eosin (HE) staining of the right lower lung was performed, and the following indices were observed under a light microscope: changes in the alveolar septum of the rats, degree of inflammatory cell infiltration, congestion and edema of pulmonary capillaries, and other histomorphological changes. The pathological conditions of lung tissues in each group were observed under a microscope through tissue section, conventional dewaxing, hematoxylin staining, color separation, dehydration, and film sealing. Pathological sections of lung tissue and HE staining were performed at the Pathology Department of Dongzhimen Hospital of Beijing University of Chinese Medicine.

### 2.7 Parallel reaction monitoring (PRM) analysis

The right lung accessory lobe of rats in each group was subjected to trypsin enzymolysis. The polypeptide concentration was determined using the bicinchoninic acid method. Differential proteins were selected and screened for PRM polypeptides using Skyline 3.6 software. Eighteen samples were verified separately, and each was analyzed with the schedule mode for the polypeptide to be verified. Independent retention time standard peptide analysis was added to each sample, and technical replicates were performed twice for each sample. Different groups of samples were mixed and interspersed with mass spectrometry to reduce systematic errors. In the Skyline software, the correct peak was manually selected, polypeptide results of all samples were derived, and then quantitative analysis was conducted.

### 2.8 Inflammatory pathway factors were detected by enzyme-linked immunosorbent assay (ELISA)

After centrifugation at 3,000 rpm for 20 min, the supernatant of the bronchoalveolar lavage fluid (BALF) was collected and stored in a − 80°C refrigerator. As detected by ELISA according to instruction, the coated microbodies, which were previously coated with protein antibodies, were sequentially added with specimens, standards, and labeled detection antibodies, and then they were incubated and thoroughly washed. The absorbance was measured at 450 nm using a microplate reader to determine the sample concentration.

### 2.9 Statistical analysis

Data are presented as mean ± standard error or standard deviation, as indicated in the legends. Data processing was performed using the SPSS 20.0 statistical software package. Statistical differences were determined by one-way analysis of variance or Mann-Whitney U-test using SPSS version 22. Differences were considered significant at P < 0.05.

## 3 Results

SEW is a type of functional water treated by an ultralow-frequency magnetic field, which is safe and nontoxic and can improve the body’s immunity. As a type of physical therapy, FIR can improve local blood circulation, strengthen tissue metabolism, promote swelling subsidence, and treat inflammatory diseases. Our results reveal that SEW and FIR can reduce ARDS in rats inflicted with LPS. Therefore, to comprehensively analyze the action of SEW and FIR treatment, we used proteomics data combined with in vivo experiments to verify the mechanism of SEW and FIR in preventing and treating LPS-induced ARDS in rats. DEPs from the SEW and FIR intervention were obtained from a proteomics database. Enrichment analysis of DEPs was performed using DAVID. The PPI network was set up using the STRING database. Modular analysis was performed using MCODE from the Cytoscape software. Tail vein injection of LPS was used to establish the ARDS rat model. PRM confirmed the associated candidate proteins. Moreover, the protein expression of related pathways of biomarkers was detected by ELISA.

### 3.1 SEW and FIR affected protein expression in lung tissue of rats with ARDS

**Figure 2.**
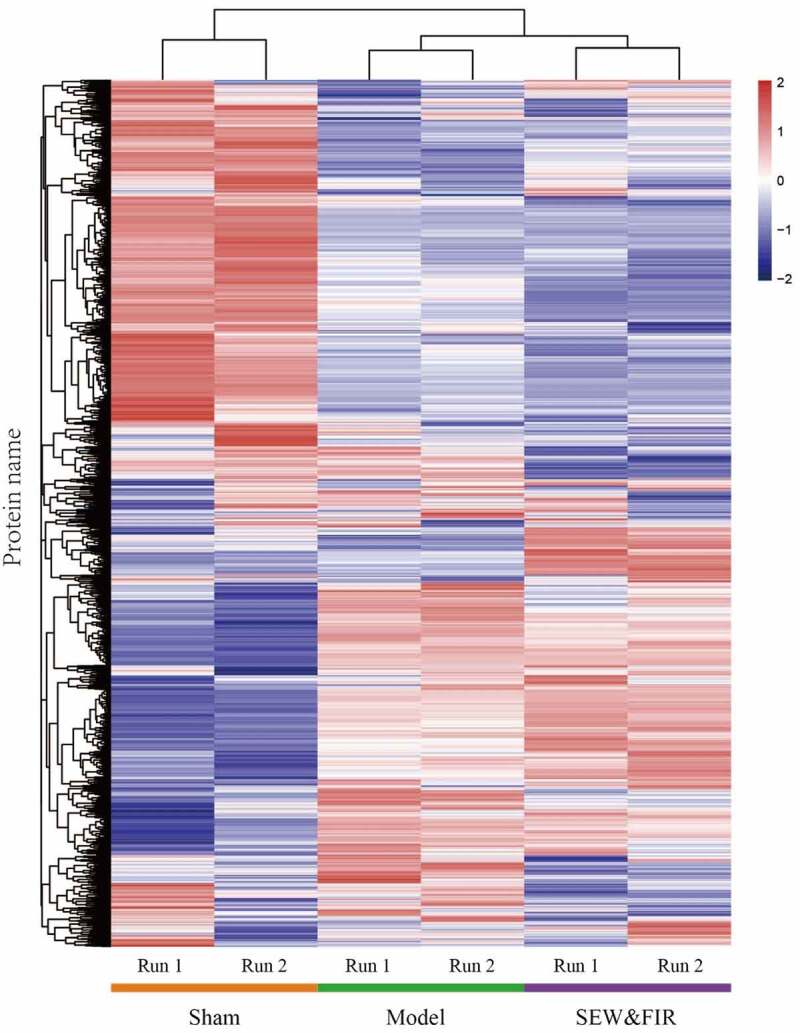
A cluster heat map of each protein identified by two repeated tandem mass tag labeling

**Figure 3.**
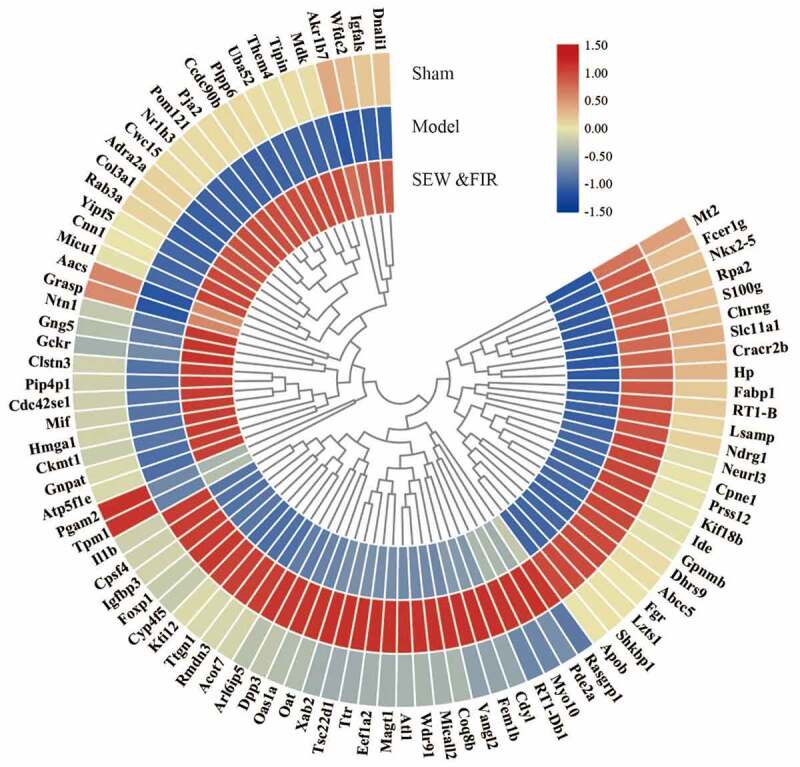
Upregulation (red) and downregulation (blue) of protein expression in the circle heat map of 89 DEPs

A total of 2,786 credible proteins were identified and quantified by two repeated tandem mass tag labeling experiments. Cluster thermogram results showed that no significant differences in protein expression were observed in the same groups, but a significant difference was observed between the groups (Figure 2). Among them, 35 proteins decreased in the model group compared with that in the sham group (model/sham <0.833) and increased in the SEW and FIR group compared with that in the model group (SEW and FIR/model >1.1), and 54 proteins increased in the model group compared with that in the sham group (model/sham >1.2) and increased in the SEW and FIR group compared with that in the model group (SEW and FIR/model <0.909), whereas a total of 89 DEPs, recovered by SEW and FIR, were changed significantly in the model group compared with that in the sham group (Figure 3). To clearly identify the names of the DEPs, TBtools were used to display a circle heat map [15].

### 3.2 GO enrichment analysis of DEPs

**Figure 4.**
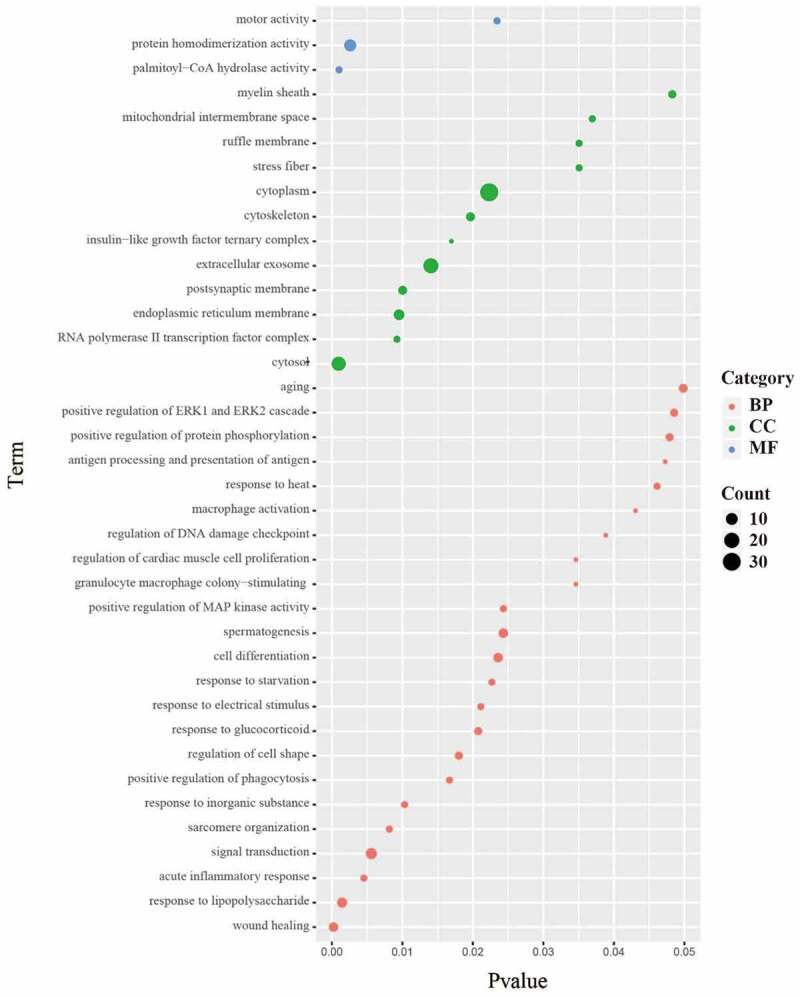
The 23 biological processes, 12 cell components, and three molecular functions. The size of the dot represents the number of genes contained in the GO function; the color represents the P value. The redder the color, the smaller the P value, and the greener the color, the larger the P value

GO function annotation and enrichment (Biological process (BP), Cellular component (CC), Molecular function (MF)) analysis of DEPs in lung tissues of rats in SEW and FIR group and model group revealed that the differential proteins were involved in 38 biological functions (P < 0.05) and 23 biological processes (P < 0.05), including: wound healing, response to LPS, acute inflammatory response, signal transduction, sarcomere organization, response to inorganic substance, positive regulation of phagocytosis, regulation of cell shape, response to glucocorticoids, and response to electrical stimulus. 12 cell components were enriched (P < 0.05), that is, the cytosol, polymerase II transcription factor complex, endoplasmic reticulum membrane,, extracellular exosome, insulin-like growth factor ternary complex, cytoskeleton, cytoplasm, stress fiber, and ruffle membrane (Figure 4). The main functions can be summarized as wound healing, inflammatory response, sarcomere tissue function, phagocytosis and enzyme activity.

### 3.3 PPI construction and core protein selection of DEPs

**Figure 5.**
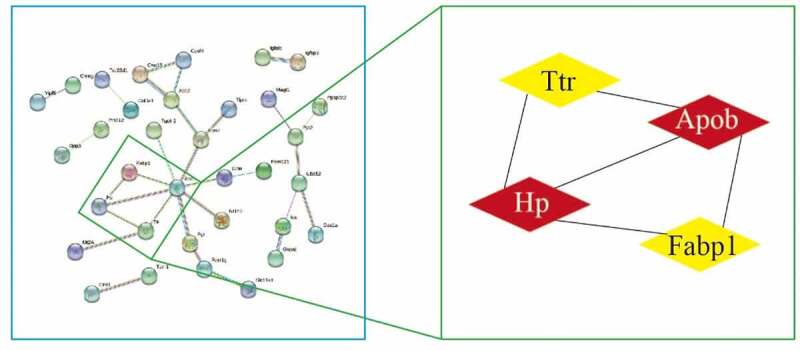
Topological screening for the PPI network

We analyzed 89 DEPs in the STRING database, set medium confidence to 0.4, hide disconnected nodes in the network, analyzed PPI using MCODE in Cytoscape across the entire network, set the degree cutoff to 2, and obtained the core network structure with the highest score. The following were obtained: score 3.333, nodes 4, edges 5, and core proteins Hp, Apob, Ttr, and Fabp1 (Figure 5).

### 3.4 SEW and FIR alleviated pathological injury of lung tissue in rats with ARDS

**Figure 6.**
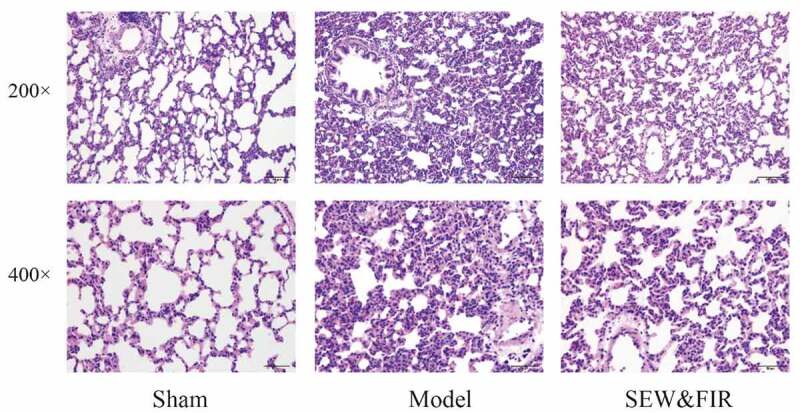
Lung tissues subjected to HE staining and observed under a light microscope at 200× and 400× magnifications. Representative images of each group are shown at scale bars of 100 μm and 50 μm. Sham indicates the sham group, model as the ARDS rat model control, and special electromagnetic field-treated water (SEW) and far-infrared radiation (FIR) as the SEW and FIR group

Compared with the sham group, rats in the model group had edema in the alveoli and pulmonary interstitium, with a large number of inflammatory cell infiltrations, thickening of alveolar walls, reduction and deformation of alveolar cavity, partial alveolar collapse, and a large amount of secretions in the bronchial and alveolar cavities, including red blood cells, congestion, and edema of pulmonary capillaries. The pathological changes in the lung tissue of rats in the SEW and FIR group were less severe than those in the model group, showing a small amount of inflammatory cell infiltration, less alveolar collapse, less bleeding, exudation, etc. (Figure 6).

### 3.5 SEW and FIR interfered with Hp-mediated acute inflammation

**Figure 7.**
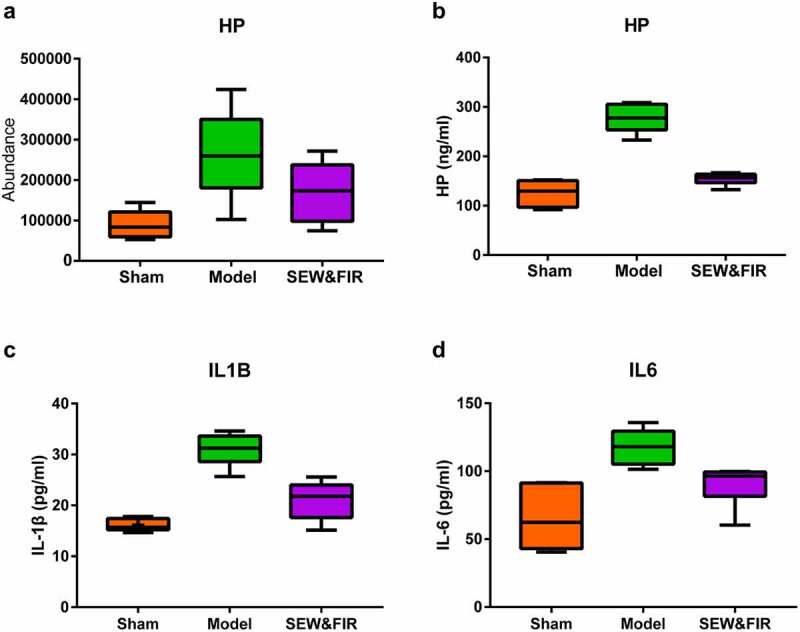
(a) The result of PRM verification. Compared with the model group, the expression of Hp was restored after treatment with SEW and FIR, n = 6. (B, C, and D) The result of ELISA. Compared with the sham group, the expression level of Hp, IL-6, and IL-1β in BALF in the model group was significantly increased (P < 0.01). Compared with the model group, the expression level of Hp, IL-6, and IL-1β in the SEW and FIR group was significantly decreased

Based on the results of proteomics, enrichment analysis and PPI, Haptoglobin (Hp) was selected for verification. PRM analysis showed that. Hp increased in model group compared with Sham group, and recovered after SEW&FIR intervention (Figure 6a). In the GO enrichment analysis, Hp mainly involved gene functions such as responses to LPS, acute inflammation, extracellular exosome, glucocorticoid, electrical stimulus, starvation, and heat (Figure S1). ELISA results also showed that compared with the sham group, the expression level of Hp in BALF in the model group was significantly increased (P < 0.01). Compared with the model group, the expression level of Hp in the SEW and FIR group was significantly decreased (P < 0.01) (Figure 7). Compared with the sham group, the expression level of IL-1β in BALF was significantly increased in the model group (P < 0.01). Compared with the model group, the expression level of IL-1β in the SEW and FIR group was significantly decreased (P < 0.01) (Figure 6c). Compared with the sham group, the expression level of IL-6 in BALF was significantly increased in the model group (P < 0.01). Compared with the model group, the expression level of IL-6 in the SEW and FIR group was significantly decreased (P < 0.05) (Figure 6d).

## 4 Discussion

ARDS is mainly treated with protective mechanical ventilation and restrictive fluid management to support the treatment and control the primary disease. There are no special treatment methods or drugs to regulate excessive immune responses (inhibit uncontrolled activation of macrophages and neutrophils) and protect and reduce extensive endothelial barrier damage. Some drugs used in clinics, such as glucocorticoids, have uncertain prognoses due to differences in the timing, dosage, and target [16]. The curative effect and prognosis of extracorporeal membrane oxygenation are also unstable, and the treatment risks and costs are extremely high [17,18]. Stem cells can reduce lung injury and enhance lung tissue repair with certain therapeutic potential, but effective clinical evidence is still lacking [19,20]. Drugs such as β2-agonists, statins, and keratinocyte growth factors have achieved certain therapeutic effects in animal experiments, but they have not achieved satisfactory effects in clinical applications and have serious clinical side effects [21–23]. A study on transcriptome analysis predicts drug candidates for sepsis-induced ARDS; doxorubicin could be a potential therapeutic for sepsis-induced ARDS by targeting TOP2A. However, this is only a statistical analysis and requires further investigation and validation [24]. Therefore, a safe and effective treatment method based on an in-depth discussion of the pathogenesis of ARDS is urgently needed.

SEW and FIR, as a physical intervention factor, can reduce the pathological damage of lung tissue in LPS-induced ARDS in model rats and reduce the expression of inflammatory cytokines in serum and inflammatory pathway proteins in the lung tissue. SEW is a small molecular water, which has the capacity to change its chemical properties, such as the pH value and ions. Many chemical reactions in the human body occur when water is used as a solvent, and as infrared intervention matches the human body frequency, the thermal and electromagnetic effects of FIR also affect the properties of proteins in the human body, which is a multitarget regulation. FIR is a form of thermal radiation that may have beneficial effects on cardiovascular health. Animal studies have shown that far-infrared mechanisms include the activation of oxidative stress and heat shock proteins. Clinical studies have shown that FIR may have therapeutic effects on heart failure and myocardial ischemia and may improve the flow rate and survival rate of arteriovenous fistulas [25], implying that FIR may have a certain recovery effect on inflammation injury in living organisms. Proteomics research methods were adopted to further explain the mechanism of the SEW and FIR intervention.

Experimental data showed that after the SEW and FIR intervention in model rats, there were 89 DEPs, of which 54 were upregulated and 39 were downregulated. The enrichment analysis of the functional annotation of GO proteins differentially expressed in lung tissues of rats in the SEW and FIR and model groups revealed 38 biological functions, including 23 biological processes, 12 cellular components, and three molecular functions. The main functions include wound healing, inflammatory response, sarcomere tissue function, phagocytosis, and enzyme activity. This echoes previous research. Bioceramic is a type of FIR that can launch high-performance materials and has been shown to have certain biological effects in vivo and in vitro, including amphibian movement in muscle cells against oxidative stress and prevention of the anti-inflammatory analgesic mechanism of skeletal muscle fatigue. Positron emission tomography monitoring under LPS injection has been used in rabbits with inflammatory arthritis [26]. Infrared rays have a stimulatory effect on skin wound healing [27]. Among the various proteins, the network nodes that have core functions have been screened out, verifying the main function of the Hp gene. Hp is a conservative protein synthesized mainly in the liver and lungs and is a potential biomarker of many diseases [28]. Hp, as an antioxidant, has antibacterial properties and plays multiple roles in modulating the acute phase response. Hp is a rich hemoglobin-binding protein present in the plasma. It primarily determines the fate of hemoglobin after intravascular or extravascular hemolysis of red blood cells [29]. Furthermore, Hp is related to the development of several diseases, like cardiovascular disease, psoriasis, chronic pancreatitis, Parkinson, and myelofibrosis [30–34].

**Figure 8.**
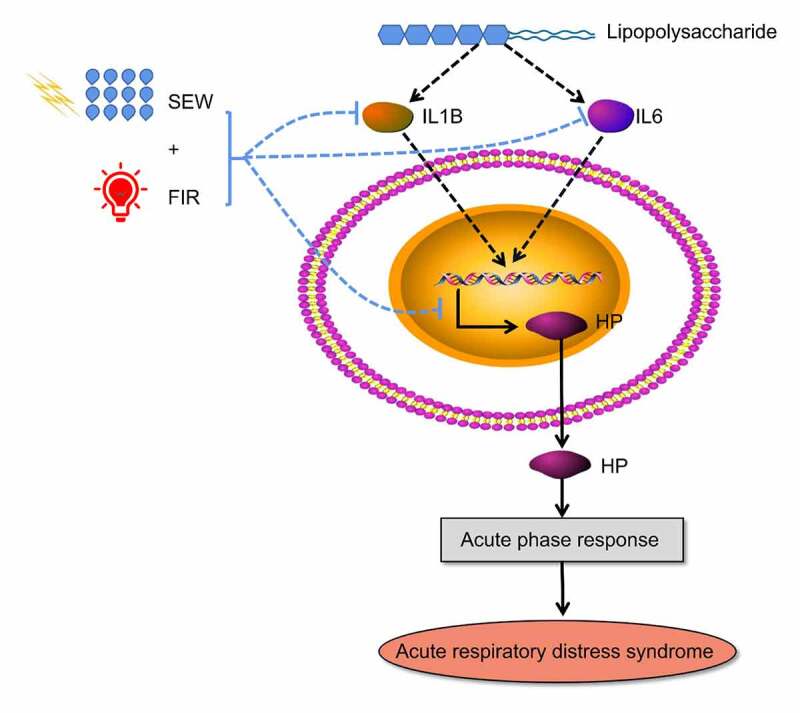
Possible regulatory mechanisms of SEW and FIR to alleviate ARDS

Hp participates in the enrichment of differential proteins, including LPS and acute inflammatory response, which indicates that SEW and FIR can alleviate the systemic inflammatory response caused by LPS, especially in the lungs, by regulating Hp reduction. In summary, LPS activated immune cytokines (IL-1B, IL6), induced the expression of HP, and further induced acute inflammatory response, and then caused the Acute respiratory distress syndrome. SEW & FIR could reduced the expression and immune cytokines (IL-1B, IL6) and HP, and alleviate systemic inflammatory response caused by LPS (Figure 8). Additionally, SEW and FIR may also play a role in regulating biological functions such as responses to extracellular exosome, electrical stimulus, and heat through Hp. Although physical factors cannot be used as drugs to directly combine with the target protein of the disease, they can change the occurrence of chemical reactions by changing the properties of solvents and changes in temperature and magnetic fields; thus, they can change the expression of related proteins to play a role in intervening diseases. Our results suggest that SEW and FIR are potential treatments for ARDS. Current studies show that the therapeutic effect of SEW and FIR is mainly to reduce the inflammatory response, possibly through the regulation of the Hp protein. The effect of SEW and FIR and the differential analysis of lung tissue protein expression were verified only by animal experiments at the lung tissue level, and its mechanism of action could be further explored through cell experiments.

## 5 Conclusion

In conclusion, SEW and FIR can improve pathological hemorrhage and edema in the lung tissue of rats with ARDS induced by LPS and reduce inflammatory cell exudation. We speculate that the protective effect of SEW and FIR on endotoxin-induced ARDS may result from their role in regulating the Hp proteins, which are associated with the acute inflammatory response.

## Supplementary Material

Supplemental Material

## Data Availability

Publicly available datasets were analyzed in this study. These data can be found in the repository iProX, with accession ID IPX0002375000 (http://www.iprox.org).

## Abbreviations

ARDS, acute respiratory distress syndrome; NF-κB, nuclear factor kappa-B; TNF-α, tumor necrosis factor-α; LPS, lipopolysaccharide; IL, interleukin; DEPs, differentially expressed proteins; PRM, parallel reaction monitoring; PPI, protein-protein interaction; GO, Gene Ontology; SEW, special electromagnetic field-treated water; FIR, far-infrared radiation; DAVID, Database for Annotation, Visualization and Integrated Discovery; BALF, Bronchoalveolar lavage fluid; ELISA, enzyme-linked immunosorbent assay

## References

[1] Thompson BT, Chambers RC, Liu KD. Acute respiratory distress syndrome. N Engl J Med. 2017;377(6):562–572. 28792873 10.1056/NEJMra1608077

[2] Fan E, Brodie D, Slutsky AS. Acute respiratory distress syndrome: advances in diagnosis and treatment. JAMA. 2018;319(7):698–710. 29466596 10.1001/jama.2017.21907

[3] Meyer NJ, Gattinoni L, Calfee CS. Acute respiratory distress syndrome. Lancet. 2021 Jul 1:S0140;6736(21):00439. 10.1016/S0140-6736(21)00439-6 PMC8248927 34217425

[4] Ma JW, Li QQ, Ji DD, et al. Predicting candidate therapeutic drugs for sepsis-induced acute respiratory distress syndrome based on transcriptome profiling. Bioengineered. 2021;12(1):1369–1380. 33904373 10.1080/21655979.2021.1917981 PMC8806268

[5] Yang CY, Chen CS, Yiang GT, et al. New insights into the immune molecular regulation of the pathogenesis of acute respiratory distress syndrome. Int J Mol Sci. 2018;19(2):588. 10.3390/ijms19020588 PMC5855810 29462936

[6] Meduri GU, Bridges L, Siemieniuk RAC, et al. An exploratory reanalysis of the randomized trial on efficacy of corticosteroids as rescue therapy for the late phase of acute respiratory distress syndrome. Crit Care Med. 2018;46(6):884–891. 29432350 10.1097/CCM.0000000000003021

[7] Xun S. Increased dielectric constant in the water treated by extremely low frequency electromhnetic field and its possible biological implication. J Phys Conf S. 2011.

[8] Liang XY, Yuan LM, Shi TX. Safety and health function evaluation of Special Electromagnetic Field Treated Water. Public Health China. 2002;4:25–26.

[9] Li J, Fan LW, Xie Y, et al. Clinical observation on 92 cases of knee osteoarthritis treated by comprehensive therapy. Chin Foreign Med Treat. 2012;31(28):91+93.

[10] Toyokawa H, Matsui Y, Uhara J, et al. Promotive effects of far-infrared ray on full-thickness skin wound healing in rats. Exp Biol Med (Maywood). 2003;228(6):724–729. 12773705 10.1177/153537020322800612

[11] Li Y, Wang S, Li X, et al. Impact of Yiqi Huayu Jiedu Decoction combined with ultra-low frequency electromagnetic field treated water on endotoxin-induced acute lung injury in rats. J Tradit Chin Med. 2016;57:783–788.

[12] Huang R, Li Y, Li X, et al. Study on the Protective Effect of Special Electromagnetic Field Treated Water and Far Infrared Rays on LPS-Induced ARDS Rats. Evid Based Complement Alternat Med. 2019;2019:1–7. 10.1155/2019/5902701 PMC6590563 31281400

[13] Luo CY, Li Y, Li X, et al. Effects of Yiqi Huayu Jiedu decoction and spectrum water on serum IL-4 and IL-10 expressions in LPS-induced ARDS rats. J Emerg Tradit Chin Med. 2019;28:1430–1434.

[14] Luo CY, Li Y, Li X, et al. Alleviation of lipopolysaccharide-induced acute respiratory distress syndrome in rats by Yiqi Huayu Jiedu decoction: a tandem mass tag-based proteomics study. Front Pharmacol. 2020;11:1215. 32982719 10.3389/fphar.2020.01215 PMC7485520

[15] Chen C, Chen H, Zhang Y, et al. TBtools: an integrative toolkit developed for interactive analyses of big Biological Data. Mol Plant. 2020;13(8):1194–1202. 32585190 10.1016/j.molp.2020.06.009

[16] Williams GW, Berg NK, Reskallah A, et al. Acute respiratory distress syndrome. Anesthesiology. 2021;134(2):270–282. 33016981 10.1097/ALN.0000000000003571 PMC7854846

[17] Paolone S. Extracorporeal membrane oxygenation (ECMO) for lung injury in severe acute respiratory distress syndrome (ARDS): review of the literature. Clinical Nursing Research. 2017;26(6):747–762. 27836935 10.1177/1054773816677808

[18] Barbaro RP, Xu Y, Borasino S, et al. RESTORE Study Investigators *. Does extracorporeal membrane oxygenation improve survival in pediatric acute respiratory failure? Am J Respir Crit Care Med. 2018;197(9):1177–1186. 29373797 10.1164/rccm.201709-1893OC PMC6019927

[19] Laffey JG, Matthay MA. Fifty years of research in ARDS. Cell-based therapy for acute respiratory distress syndrome. Biology and potential therapeutic value. Am J Respir Crit Care Med. 2017;196(3):266–273. 28306336 10.1164/rccm.201701-0107CP PMC5549868

[20] Guillamat-Prats R, Camprubí-Rimblas M, Bringué J, et al. Cell therapy for the treatment of sepsis and acute respiratory distress syndrome. Ann Transl Med. 2017;5(22):446. 29264363 10.21037/atm.2017.08.28 PMC5721220

[21] Perkins GD, Gates S, Park D, et al. The beta agonist lung injury trial prevention. A randomized controlled trial. Am J Respir Crit Care Med. 2014;189(6):674–683. 24392848 10.1164/rccm.201308-1549OC PMC3983838

[22] McAuley DF, Laffey JG, O’Kane CM, et al. Simvastatin in the acute respiratory distress syndrome. N Engl J Med. 2014;371(18):1695–1703. 25268516 10.1056/NEJMoa1403285

[23] McAuley DF, Cross LM, Hamid U, et al. Keratinocyte growth factor for the treatment of the acute respiratory distress syndrome (KARE): a randomised, double-blind, placebo-controlled phase 2 trial. Lancet Respir Med. 2017;5(6):484–491. 28526233 10.1016/S2213-2600(17)30171-6

[24] Fang Q, Wang QL, Zhou ZM, et al. Consensus analysis via weighted gene co-expression network analysis (WGCNA) reveals genes participating in early phase of acute respiratory distress syndrome (ARDS) induced by sepsis. Bioengineered. 2021;12(1):1161–1172. 33818300 10.1080/21655979.2021.1909961 PMC8806251

[25] Shemilt R, Bagabir H, Lang C, et al. Potential mechanisms for the effects of far-infrared on the cardiovascular system - A review. Vasa. 2019 Jul;48(4):303–312. 30421656 10.1024/0301-1526/a000752

[26] Leung TK. In vitro and in vivo Studies of the Biological Effects of Bioceramic (a Material of Emitting High Performance Far-infrared Ray) Irradiation. Chin J Physiol. 2015 Jun 30;58(3):147–155. 26014120 10.4077/CJP.2015.BAD294

[27] Yadav A, Gupta A. Noninvasive red and near-infrared wavelength-induced photobiomodulation: promoting impaired cutaneous wound healing. Photodermatology, Photoimmunology & Photomedicine. 2017 Jan;33(1):4–13. 10.1111/phpp.12282 27943458

[28] Naryzhny SN, Legina OK. Gaptoglobin kak biomarker [haptoglobin as a biomarker]. Biomed Khim. 2021 Mar;67(2):105–118. 33860767 10.18097/PBMC20216702105

[29] Levy AP, Asleh R, Blum S, et al. Haptoglobin: basic and clinical aspects. Antioxid Redox Signal. 2010;12(2):293–304. 19659435 10.1089/ars.2009.2793

[30] Graves KL, Vigerust DJ. Hp: an inflammatory indicator in cardiovascular disease. Future Cardiology. 2016 Jul;12(4):471–481. 27203711 10.2217/fca-2016-0008

[31] Tian FJ, Zhang YY, Liu LQ, et al. Haptoglobin protein and mRNA expression in psoriasis and its clinical significance. Mol Med Rep. 2016;14(4):3735–3742. 27571879 10.3892/mmr.2016.5672

[32] Ueda M, Kamada Y, Takamatsu S, et al. Specific increase in serum core-fucosylated haptoglobin in patients with chronic pancreatitis. Pancreatology. 2016;16(2):238–243. 26897254 10.1016/j.pan.2016.01.004

[33] Delanghe JR, De Buyzere ML, Speeckaert MM, et al. Haptoglobin phenotype and Parkinson disease risk. Parkinsonism Relat Disord. 2016;22:108–109. 26708125 10.1016/j.parkreldis.2015.10.020

[34] Strati P, Masarova L, Bose P, et al. Haptoglobin is frequently low in patients with myelofibrosis: clinical relevance. Leuk Res. 2017;57:85–88. 28324773 10.1016/j.leukres.2017.03.006

