# Supplementary material for: Special electromagnetic field-treated water and far-infrared radiation alleviates lipopolysaccharide-induced acute respiratory distress syndrome in rats by regulating haptoglobin
Source: Bioengineered. 2021 Sep 14;12(1):6808–20. doi: 10.1080/21655979.2021.1969201 (PMC8806454; doi:10.1080/21655979.2021.1969201)
Supplement: Supplemental Material [file KBIE_A_1969201_SM2440.zip › spp.pdf]

Figure S1

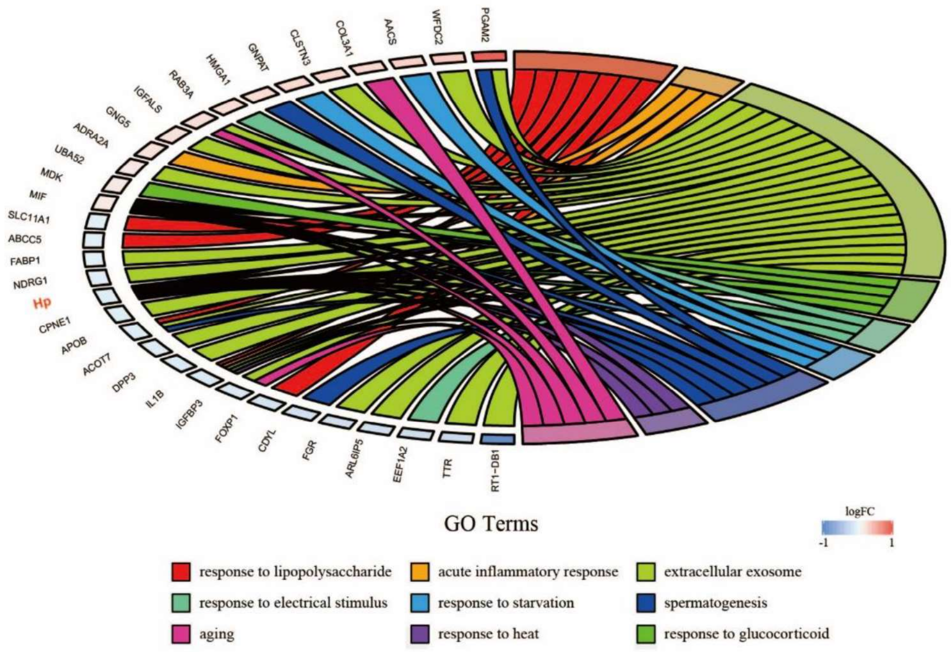
PGAM2
WFDC2
AACS
COL3A1
CLSTN3
GNPAT
HMGA1
RAB3A
IGFALS
GNG5
ADRA2A
UBA52
MDK
MIF
SLC11A1
ABCC5
FABP1
NDRG1
Hp
CPNE1
APOB
ACOT7
DPP3
IL1B
IGFBP3
FOXP1
CDYL
FGR
ARL6IP5
EEF1A2
TTR
RT1-DB1
GO Terms
logFC
-1
1
response to lipopolysaccharide
acute inflammatory response
extracellular exosome
response to electrical stimulus
response to starvation
spermatogenesis
aging
response to heat
response to glucocorticoid

Figure Legends

Figure 1. The workflow of the study

Figure 2. A cluster heat map of each protein identified by two repeated tandem mass tag labeling

Figure 3. Upregulation (red) and downregulation (blue) of protein expression in the circle heat map of 89 DEPs

Figure 4. The 23 biological processes, 12 cell components, and three molecular functions. The size of the dot represents the number of genes contained in the GO function; the color represents the P value. The redder the color, the smaller the P value, and the greener the color, the larger the P value

Figure 5. Topological screening for the PPI network

Figure 6. Lung tissues subjected to HE staining and observed under a light microscope at 200× and 400× magnifications. Representative images of each group are shown at scale bars of 100 μm and 50 μm. Sham indicates the sham group, model as the ARDS rat model control, and special electromagnetic field-treated water (SEW) and far-infrared radiation (FIR) as the SEW and FIR group

Figure 7. (A) The result of PRM verification. Compared with the model group, the expression of Hp was restored after treatment with SEW and FIR, n = 6. (B, C, and D) The result of ELISA. Compared with the sham group, the expression level of Hp, IL-6, and IL-1β in BALF in the model group was significantly increased ($P < 0.01$). Compared with the model group, the expression level of Hp, IL-6, and IL-1β in the SEW and FIR group was significantly decreased

Figure 8. Possible regulatory mechanisms of SEW and FIR to alleviate ARDS

Figure S1. The differential genes between GO and Hp Terms with the participation of Hp
